# Stimuli‐Responsive Aggregation of High Molar Mass Poly(*N*,*N*‐Diethylacrylamide)‐*b*‐Poly(4‐Acryloylmorpholine) in Tetrahydrofuran

**DOI:** 10.1002/marc.202100656

**Published:** 2021-11-25

**Authors:** Alexander Plucinski, Marko Pavlovic, Mairi Clarke, David Bhella, Bernhard V. K. J. Schmidt

**Affiliations:** ^1^ School of Chemistry University of Glasgow Glasgow G12 8QQ UK; ^2^ Department of Colloid Chemistry Max Planck Institute of Colloids and Interfaces Am Mühlenberg 1 Potsdam 14476 Germany; ^3^ BioSense Institute University of Novi Sad Dr Zorana Djindjica 1, III‐8 Novi Sad 21000 Serbia; ^4^ Scottish Centre for Macromolecular Imaging University of Glasgow Glasgow G61 1QH UK

**Keywords:** block copolymers, high molecular weight, reversible deactivation radical polymerization, self‐assembly, stimuli responsive polymers

## Abstract

The self‐assembly of block copolymers constitutes a timely research area in polymer science with implications for applications like sensing or drug‐delivery. Here, the unprecedented aggregation behavior of high molar mass block copolymer poly(*N*,*N*‐diethylacrylamide)‐*b*‐poly(4‐acryloylmorpholine) (PDEA‐*b*‐PAM) (*M*_n_>400 kg mol^−1^) in organic solvent tetrahydrofuran (THF) is investigated. To elucidate the aggregation, dynamic light scattering, cryo‐transmission electron microscopy, and turbidimetry are employed. The aggregate formation is assigned to the unprecedented upper critical solution temperature behavior of PAM in THF at elevated concentrations (> 6 wt.%) and high molar masses. Various future directions for this new thermo‐responsive block copolymer are envisioned, for example, in the areas of photonics or templating of inorganic structures.

## Graphical abstract

Aggregation behavior of high molar mass block copolymer poly(*N,N*‐diethylacrylamide)‐*b*‐poly(4‐acryloylmorpholine) in organic solvent tetrahydrofuran is investigated.

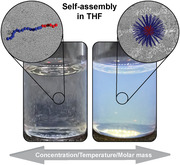

## 1 Introduction

Self‐assembly and aggregation of polymers belong to the most relevant research areas in polymer science and their application,^[^ ^1^, ^2^, ^3^ ^]^ for example, in photonic materials for sensing^[^ ^4^ ^]^ or drug‐delivery.^[^ ^5^ ^]^ An important factor for the structures formed in self‐assembly and aggregation of polymers, is the molar mass of the block copolymers. Thus, high molar mass polymers attracted attention recently.^[^ ^6^, ^7^ ^]^ A strategy in the formation of well‐defined aggregates is the utilization of block copolymer self‐assembly. In a selective solvent for one of the polymer blocks, aggregates like micelles,^[^ ^8^ ^]^ or vesicles^[^ ^9^ ^]^ are formed. Furthermore, in a non‐selective solvent, aggregates can be formed via external triggers, for example, frequently with temperature^[^ ^10^ ^]^ or pH triggers.^[^ ^11^ ^]^ In the case of a temperature trigger, aggregates are formed exploiting a lower critical solution temperature (LCST)^[^ ^12^ ^]^ or an upper critical solution temperature (UCST)^[^ ^13^ ^]^ of one of the polymer building segments in the block copolymer. For example, the LCST of poly(*N*‐isopropylacrylamide) (PNIPAM)^[^ ^12^ ^]^ as well as poly(*N,N*‐diethylacrylamide) (PDEA)^[^ ^14^ ^]^ in water are well known and exploited to form stimuli‐responsive aggregates, for example, poly(styrene)‐*b*‐PNIPAM‐*b‐*poly(styrene)^[^ ^15^ ^]^ or poly(*N,N*‐dimethylacrylamide)‐*b*‐PDEA^[^ ^16^ ^]^ upon heating the mixture.

Besides self‐assembly in aqueous environment, organic solvents and solvent mixtures are of interest as well,^[^ ^17^, ^18^ ^]^ for example, in the stabilization of oil‐in‐oil emulsions^[^ ^19^, ^20^ ^]^ or in the formation of micellar photonic crystals.^[^ ^21^ ^]^ The group of Urban formed thermochromic inverse polymeric micelles in toluene, using ultra‐high molar mass poly(2‐(*N*,*N*‐dimethylamino)ethyl methacrylate)‐*b*‐poly(*n*‐butyl acrylate).^[^ ^22^ ^]^ Gröschel and coworkers used block copolymer micelle formation in combination with solvent exchange and following dilution to generate photonic fluids and crystals.^[^ ^23^ ^]^

One key factor for polymer properties, like self‐assembly or aggregation, is molar mass. In order to synthesize novel high molar mass block copolymers, reversible addition‐fragmentation chain transfer (RAFT) polymerization is a facile avenue.^[^ ^24^, ^25^ ^]^ Sumerlin and coworkers utilized aqueous photoiniferter‐mediated (PI) RAFT polymerization for the synthesis of high and ultra‐high molaf mass poly(acrylamides).^[^ ^26^ ^]^ At first, primarily homopolymers or chain extensions were investigated, for example, PDMA and PDMA‐*b*‐PDMA.^[^ ^26^ ^]^ Additionally, Sumerlin and coworkers utilized PI‐RAFT polymerization for the synthesis of ultra‐high molar mass block copolymers, for example, poly(*N,N*‐dimethylacrylamide)‐*b*‐poly(*tert*‐butyl acrylate), which forms assemblies upon solvent switch from THF to H_2_O.^[^ ^27^ ^]^ Such high molar mass block copolymers could find considerable applications in the fields of photonics^[^ ^21^, ^23^ ^]^ and lithography.^[^ ^28^ ^]^

**Scheme 1:**
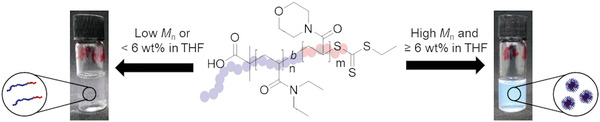
Overview of the aggregation behavior of poly(*N,N*‐diethylacrylamide)‐*b*‐poly(4‐acryloylmorpholine) (PDEA‐*b‐*PAM) in THF, depending on molar mass or concentration.

Herein, we present the unprecedented temperature‐responsive aggregation of the high molar mass block copolymer PDEA‐*b*‐poly(4‐acryloylmorpholine) (PDEA‐*b*‐PAM) in the organic solvent tetrahydrofuran (THF). The aggregation process leads to large particles with sizes in the range of 100 to 260 nm (**Scheme** **1**). Analysis at different concentrations and temperatures via dynamic light scattering (DLS), cryo‐(transmission electron microscopy) (cryo‐TEM), and temperature‐controlled UV–vis spectroscopy revealed the condition for aggregation being elevated concentration (6 wt.%) and high block copolymer molar mass.

## 2 Results and Discussions

**Figure 1:**
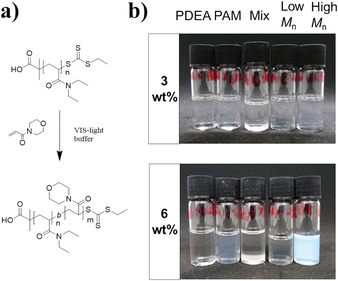
a) Reaction scheme of PDEA‐*b*‐PAM formation via visible light photo‐induced RAFT‐polymerization, b) solubility behavior of PDEA_1850_, PAM_830_, PDEA_1850_/PAM_830_ mix, PDEA_98_‐*b‐*PAM_387_, and PDEA_1850_‐*b‐*PAM_1380_ in THF at 3 and 6 wt.%.

PDEA‐*b*‐PAM was synthesized via RAFT polymerization. At first, the PDEA block was synthesized, yielding PDEA with a molar mass of *M*_n_ = 203 000 g∙mol^−1^ and a molecular dispersity of (*Ð)* of 1.3 according to SEC‐MALS (Figure S1c and Table S1, Supporting Information). Based on the literature,^[^ ^26^ ^]^ the block copolymer was synthesized via visible light‐mediated PI‐RAFT polymerization of AM in a highly concentrated buffer solution (**Figure** **1**a). PDEA‐*b*‐PAM was obtained with a molar mass of *M*_n_ = 403 000 g mol^−1^ and *Ð* of 1.5 according to SEC‐MALS. The increment of absolute molar mass (Figure S1c, Supporting Information) and the signals for both polymers, around 3.5 ppm for PAM and around 3.0 ppm for PDEA, in the ^1^H‐NMR (Figure S4, Supporting Information), confirm the successful formation of the block copolymer. Additionally, the synthesis of the high molar mass block copolymer was verified via diffusion‐ordered NMR spectroscopy (DOSY) that revealed signals at a diffusion coefficient of 7.5 10^−7^ cm^2^ s^−1^ for both block types (Figure S5, Supporting Information). Moreover, the block copolymer formation was supported via differential scanning calorimetry (DSC), as observed by two glass transition temperatures (*T*_g_) corresponding to the individual polymer blocks (Figure S6 and Table S2, Supporting Information).

Unexpectedly, PDEA_1850_‐*b‐*PAM_1380_, forms blue dispersions at high concentrations in THF, that is, above 6 wt.% (Figure 1b). To verify the influence of the molar mass and to compare the block copolymer to the homopolymers, solubility behavior of PDEA_1850_, PAM_830_, PDEA_1850_/PAM_830_ mix, PDEA_98_‐*b‐*PAM_387_, and PDEA_1850_‐*b‐*PAM_1380_ was analyzed at 3 and 6 wt.% in THF. A color change could be observed only for the PDEA_1850_‐*b*‐PAM_1380_ above 6 wt.% (Figure 1b) indicating aggregation of the block copolymer in THF.

**Figure 2:**
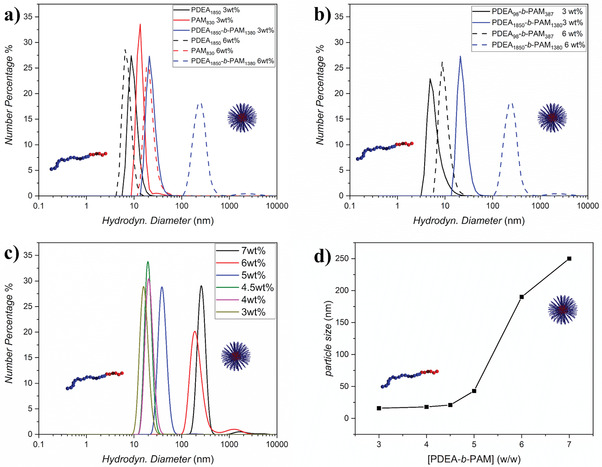
a) Comparison of number weighted particle size distribution of PDEA_1850_ (black curve), PAM_830_ (red curve), and PDEA_1850_‐*b‐*PAM_1380_ (blue curve) at different concentration, measured in THF at ambient temperature, b) PDEA_98_‐*b‐*PAM_387_ (black curve) and PDEA_1850_‐*b‐*PAM_1380_ (blue curve) at different concentration measured in THF at ambient temperature, c) number weighted particle size distribution of PDEA_1850_‐*b‐*PAM_1380_ at different concentration measured in THF at ambient temperature, and d) particle size change at different concentration in THF at ambient temperature.

In order to analyze the aggregation behavior of PDEA_1850_‐*b‐*PAM_1380_ in THF, PDEA_1850_, PAM_830_, PDEA_98_‐*b‐*PAM_387_, and PDEA_1850_‐*b‐*PAM_1380_ were dissolved in THF at different concentrations (3 and 6 wt.%). The hydrodynamic diameter was monitored for all concentrations via DLS at 25 °C (**Figure** **2**a,b). The DLS results show for all polymers at 3 wt.% a hydrodynamic diameter between 10 and 30 nm, which can be most likely assigned to free polymer chains in solution. The difference in the hydrodynamic diameter at 3 wt.% can be explained by the different molar masses of the respective polymers. At a concentration of 6 wt.%, the hydrodynamic diameter was in a similar range (around 8 to 30 nm) for both homopolymers (PDEA_1850_ and PAM_830_) and the low molar mass block copolymer PDEA_98_‐*b‐*PAM_387_ (10 to 20 nm). In contrast, the hydrodynamic diameter of PDEA_1850_‐*b‐*PAM_1380_ increased significantly to 230 nm at 6 wt.%, confirming the presence of aggregates for the high molar mass block copolymer in THF at a higher concentration. Thus, the concentration dependence of the aggregation of PDEA_1850_‐*b‐*PAM_1380_ in THF was further analyzed via DLS between 3 and 7 wt.% (Figure 2c). The concentration‐dependent DLS measurement shows that PDEA_1850_‐*b‐*PAM_1380_ has a critical aggregation concentration between 5 and 6 wt.%. As such, the turbidity of the dispersion can be explained by the high molar mass of the block copolymer leading to the formation of large aggregates and scattering of light.

**Figure 3:**
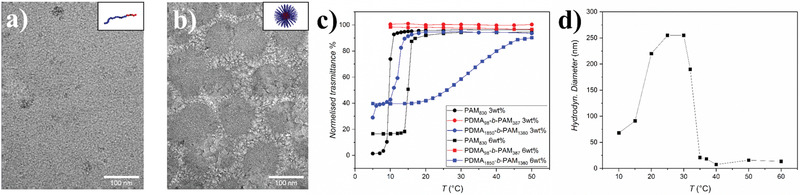
a,b) Cryo‐TEM images of PDEA‐*b*‐PAM in THF at a concentration of 6 wt.% (frozen from 25 °C) (a) PDEA_98_‐*b‐*PAM_387_ and (b) PDEA_1850_‐*b‐*PAM_1380_. c) Cooling curves of PAM_830_ (black curves), PDEA_98_‐*b‐*PAM_387_ (red curves) and PDEA_1850_‐*b‐*PAM_1380_ (blue curves) measured via turbidimetry in THF, d) particle size change of PDEA_1850_‐*b‐*PAM_1380_ aggregates at 6 wt.% in THF at different temperatures.

To further characterize the formed aggregates, the block copolymers were analyzed via cryo‐TEM. Two samples were analyzed, one block copolymer with lower molar mass (PDEA_98_‐*b‐*PAM_387_) and one with higher molar mass (PDEA_1850_‐*b‐*PAM_1380_) at a concentration of 6 wt.%. The cryo‐TEM image of PDEA_98_‐*b‐*PAM_387_ (**Figure** **3**a) displays no visible aggregation at a magnification of 50k. In contrast, the cryo‐TEM image of PDEA_1850_‐*b‐*PAM_1380_ shows aggregates with sizes between 80 and 120 nm (Figure 3b) and an average particle size of 105 ± 15 nm (Figure S7, Supporting Information). The results of the cryo‐TEM measurement confirm the molar mass influence of PDEA‐*b‐*PAM on the formation of aggregates in THF. In comparison to the DLS measurement the particle size in the cryo‐TEM images is slightly lower. The difference could be due to imaging of denser aggregate cores in cryo‐TEM compared to the full particle including corona in DLS.

After analysis of the formed aggregates, the underlying driving force was investigated. It was noticed that the aggregation of PDEA_1850_‐*b‐*PAM_1380_ depends on the temperature and is related to an UCST of PAM in THF. For example, the intensity of the blue color of the dispersion increases at lower temperatures (Figure S8, Supporting Information). In order to analyze the UCST of PAM in THF, the cloud point (*T*_cp_) of PAM_830_ and PDEA_1850_‐*b‐*PAM_1380_ were measured via turbidimetry (Figure 3c). At 3 wt.%, the *T*_cp_ was around 10 °C for PAM and between 10 and 12 °C for the block copolymer. The increase of the *T*_cp_ can be explained by the change in the phase separation process in the block copolymer, which is revealed by a broader phase transition range (10–12 °C). The effect can be explained by the presence of the stabilizing THF‐soluble PDEA blocks. As expected, the exhibited *T*_cp_ increased by 5 °C for PAM at 6 wt.%. In the case of PDEA_1850_‐*b‐*PAM_1380_ no explicit *T*_cp_ was detected but rather a transition range from 20 to 40 °C. The change in transmittance was significantly broader in comparison to PAM at 6 wt.% and both polymers at 3 wt.%, which is due to the presence of the PDEA block hindering the formation of large aggregates and a sudden aggregation via steric stabilization. It should be noted that the gradual change in transmittance was not depending on cooling time. Additionally, the particle size depends on the temperature as shown in DLS measurements of PDEA_1850_‐*b‐*PAM_1380_ at a concentration of 6 wt.% (Figure 3d and Figure S9, Supporting Information). Above 40 °C the particle size is around 15 nm which is similar to the free chain polymer at 3 wt.%. In the range of 20 to 32 °C the particle size stabilizes around 190–260 nm and the hydrodynamic diameter decreases to 70 nm between 10 and 20 °C. As such, the DLS results (Table S3, Supporting Information) for PDEA_1850_‐*b‐*PAM_1380_ in THF confirm the result of the turbidimetry that aggregation starts around 40 °C. Overall, the temperature‐dependent measurements show that the aggregation of PDEA_1850_‐*b‐*PAM_1380_ is due to an UCST of the PAM block in THF. Interestingly, aggregate formation strongly depends on concentration and molar mass. The initially observed aggregates at ambient temperature and high concentration are formed due to the presence of the UCST at temperatures under 40 °C, which leads to a blue colored dispersion, in contrast to homo polymer and low molar mass block copolymer.

## 3 Conclusions

We described the UCST behavior of PAM in THF, which was further utilized to form thermo‐responsive block copolymer aggregates via the high molar mass block copolymer PDEA_1850_‐*b‐*PAM_1380_. The formed aggregates feature a particle size of around 200 nm in highly concentrated THF solution leading to a blue colored dispersion. The aggregation depends on molar mass and concentration of the block copolymer as well as temperature. We envision various applications for this new thermo‐responsive high molar mass block copolymer, for example, in the areas of sensing, photonics, or templating of inorganic structures.

## Conflict of Interest

The authors declare no conflict of interest.

## Supporting information

Supporting Information

## Data Availability

The data that support the findings of this study are available from the corresponding author upon reasonable request.
