# Supplementary material for: Stimuli‐Responsive Aggregation of High Molar Mass Poly(N,N‐Diethylacrylamide)‐b‐Poly(4‐Acryloylmorpholine) in Tetrahydrofuran
Source: Macromol Rapid Commun. 2021 Nov 25;43(3):2100656. doi: 10.1002/marc.202100656 (PMC11475301; doi:10.1002/marc.202100656)
Supplement: Supplementary file 1 — Supporting Information [file MARC-43-2100656-s001.pdf]

Supporting Information

Stimuli-Responsive Aggregation of Poly(*N,N*-diethylacrylamide)-*b*-poly(4-acyloylmorpholine) in Tetrahydrofuran

*Alexander Plucinski,[a] Marko Pavlovic,[b,d] Mairi Clarke,[c] David Bhella[c] and Bernhard V. K. J. Schmidt[a,]**

a: School of Chemistry, University of Glasgow, Glasgow G12 8QQ, UK

b: Department of Colloid Chemistry, Max Planck Institute of Colloids and Interfaces, Am Mühlenberg 1, 14476 Potsdam, Germany.

c: Scottish Centre for Macromolecular Imaging, University of Glasgow, Glasgow G61 1QH, UK

d: BioSense Institute, University of Novi Sad, Dr Zorana Djindjica 1, 21000 Novi Sad, Serbia

Email: bernhard.schmidt@glasgow.ac.uk

Table of Contents

Section	Page
1. Experimental Section	2
2. Polymer analytics	6
3. Aggregation analytics	13

1. Experimental Section

Materials

Acetone (Fisher, analytical grade), acetic acid (1.0 N, VWR) 4-acryloylmorpholine (AM, 98%, Sigma-Aldrich, passed over a column of basic aluminium oxide), dichloromethane (DCM, analytical grade, VWR), 2-bromisobutyric acid (98.5%, pure, Sigma Aldrich), carbon disulfide (CS_2, 99%, Sigma-Aldrich), dimethyl sulfoxide (DMSO; Merck Millipore, Emsure®, ACS), *N*,*N*-dimethyl formamide (DMF, SLS) *N*,*N*-diethylacrylamide (DEA, > 98%, TCI, passed over a column of basic aluminium oxide), ethanethiol (98%, Alfa Aesar), ethyl acetate (99.5%, VWR), n-hexane (95%, Sigma-Aldrich), hydrochloric acid (conc., Fisher), Millipore water (obtained from an Sartorius Arium pro ultrapure water system), potassium phosphate (K_3PO_4, Sigma Aldrich), sodium acetate (anhydrous, 98 %, Fisher), sodium sulfate (anhydrous, SLS) and tetrahydrofuran (THF, 99.85 %, Acros Organics) were used as received unless otherwise noted. 2-(((Ethylthio)carbonothioyl)thio)-2-methylpropanoic acid (EMP) was synthesised according to the literature.[29, 30]

Photo induced RAFT (PI-RAFT) polymerization was initiated with two 50 W LED chips (Foxpic High Power 50 W LED Chip Bulb Light DIY White 3800LM 6500 K).

Analytical methods

^{1}H-NMR spectra were recorded in deuterium oxide (D_2O, Aldrich) and DMSO-d_6 at ambient temperature at 400 MHz with a Bruker Ascend400. Size exclusion chromatography (SEC) of PAM was conducted in NMP and 0.005 mol · L^{-1} LiBr with methyl benzoate as internal at 70 °C using a column system with a PSS GRAM VS; PSS GRAM 7 µm 100 A; PSS GRAM 7 mm, 1000 A and PSS SECurity Refractive Index-1260 RID and calibration with polystyrene (PS) standards. Size exclusion chromatography (SEC) of $PDEA_{98}$ and $PDEA_{98}$-*b*-PAM_{387} were conducted in THF at 35 °C using a column system with an Agilent PL Gel Guard Column (5 µm) and an Agilent PL Gel Mixed-D Column (5 µm) as well as an Agilent Infinity1260 II RID and calibration with PS standards. SEC of $PDEA_{1850}$ and $PDEA_{1850}$-*b*-PAM_{1380} was conducted in THF at 25 °C using a PSS SD guard column, a PSS SDV-Linear-M column, Wyatt Optilab DSP RI detector and a Wyatt DAWN EOS detector. A Brookhaven differential refractometer was used for the determination of d*n*/d*c*. Differential Scanning Calorimetry (DSC) was measured on a DSC 204 by Netzsch in the range from -100 °C to 220 °C. The results

from the second cycle were used for data evaluation. Cloud point (T_{cp}) measurements were performed with a Shimadzu UV-3600 UV-Vis-NIR spectrometer and a Shimadzu TCC-100 temperature-controlled cell holder. Sample in glass cuvette was placed in the sample holder and equilibrated and held at 50 °C for 5 min. Afterwards, the samples were cooled down manually in 5 or 2 °C steps and held at each temperature for 2 min. Over the entire time, transmittance at 450 nm was recorded and plotted as a function of temperature. T_{cp} was determined as the temperature at which samples exhibit half of the initial transmittance. Dynamic light scattering (DLS) was performed on a ZetaSizer by Malvern with THF as solvent. All experiments were performed three times and an average size distribution was calculated based on number weighting. Cryo-transmission electron microscopy (cryo-TEM) was performed under following conditions: 3.6 µL of polymer solution were loaded onto freshly glow-discharged Quantifoil 1.2/1.3 holey carbon support film, grids were blotted for 3 seconds and plunged into a bath of liquid nitrogen cooled liquid ethane. Specimen vitrification was performed in a Vitrobot Mark 4 from Thermo Fisher held at 22°C and 95% humidity. Vitrified samples were held in a Gatan 626 cryostage and imaged in a JEOL F200 cryo transmission electron microscope equipped with a Direct Electron DE20 detector. Images were recorded at an accelerating voltage of 200 keV.

Synthesis of 2-(((ethylthio)carbonothioyl)thio)-2-methylpropanoic acid (EMP)

Based on the literature,[1,2] ethanethiol (2.2 mL, 29.74 mmol, 1 eq.) was dissolved in a suspension of K_3PO_4 (7.46 g, 32.71 mmol, 1.1 eq.) in acetone (80 mL) at ambient temperature. After stirring for 20 min, carbon disulfide (5.4 mL, 89.22 mmol, 3.0 eq.) was added and the solution turned yellow. 2-Bromisobutyric acid (5.46 g, 32.69 mmol, 1.1 eq.) was added after 20 min and the mixture stirred at ambient temperature for 24 hours. 1 M hydrochloric acid (200 mL) was added and the aqueous phase was extracted with DCM (3 x 100 mL). The combined organic extracts were washed with deionized water (100 mL), brine (100 mL) and dried over Na_2SO_4. After evaporation of the solvent, the orange oil was purified over a column with silica gel and an eluent mixture of n-hexane: ethyl acetate 2:1. The yellow fractions were combined and the evaporation of the solvent turned the product into orange crystals (4.02 g, 17.9 mmol, 62%).

^{1}**H-NMR** (400 MHz, $CDCl_3$) [δ, ppm]:1.27 (t, J = 7.4 Hz, 3H), 1.66 (s, 6H), 3.23 (q, J = 7.4 Hz, 2H).

Synthesis of high molar mass poly(*N,N*-diethylacrylamide) ($PDEA_{1850}$)

In a dry, argon purged 100 mL round bottom Schlenk flask, destabilized DEA (5.0 g, 39.0 mmol, 2000 eq.), EMP (4.4 mg, 0.0197 mmol, 1.0 eq.) and AIBN (0.65 mg, 0.0039 mmol, 0.2 eq.) were mixed together with a stirring bar and sealed. The solution was degassed by three freeze-pump-thaw cycles and placed in a pre-heated oil bath (65 °C). The polymerization was stopped after 24 h. Subsequently, the polymer was dialyzed against deionized water (Spectra/Por 3500 Da). Finally, the sample was freeze-dried and a white solid (4.3 g, M_n= 235100 g · mol^{-1}, $Đ$ = 1.3) was obtained.

Synthesis of low molar mass poly(*N,N*-diethylacrylamide) ($PDEA_{98}$)

In a dry, argon purged 100 mL round bottom Schlenk flask, destabilized DEA (1.0 g, 7.9 mmol, 118.0 eq.), EMP (15.0 mg, 0.067 mmol, 1.0 eq.), AIBN (2.1 mg, 0.013 mmol, 0.2 eq.) were mixed together with a stirring bar and sealed. The solution was degassed by three freeze-pump-thaw cycles and placed in a pre-heated oil bath (65 °C). The polymerization was stopped after 24 h. Subsequently, the polymer was dialyzed against deionized water (Spectra/Por 3500 Da). Finally, the sample was freeze-dried and a white solid (0.8 g, M_n =12400 g · mol^{-1}, $Đ$ = 1.1) was obtained.

Synthesis of poly(4-acryloylmorpholine) (PAM_{830})

In a dry, argon purged 100 mL round bottom Schlenk flask, destabilized AM (1.0 g, 7.1 mmol, 1800 eq.), EMP (0.88 mg, 0.0039 mmol, 1.0 eq.), AIBN (0.13 mg, 0.0007 mmol, 0.2 eq.) and DMF (3 mL) were mixed together with a stirring bar and sealed. The solution was degassed by three freeze-pump-thaw cycles and placed in a pre-heated oil bath (60 °C). The polymerization was stopped after 24 h. Subsequently, the polymer was dialyzed against deionized water (Spectra/Por 3500 Da). Finally, the sample was freeze-dried and a white solid (0.9 g, M_n= 117300 g · mol^{-1}, $Đ$ = 1.6) was obtained.

Synthesis of low molar mass poly(*N,N*-diethylacrylamide)-*b*-poly(4-acryloylmorpholine) ($PDEA_{98}$-*b*-PAM_{387})

Destabilized AM (300 mg, 2.1 mmol, 260 eq.), PDEA (100 mg, 0.0081 mmol, 1.0 eq.) and acetate buffer (0.5 mL, 0.2 M, pH=5) were mixed together with a stirring bar in a glass vial (14 mL) and sealed with a septum. The solution was bubbled with nitrogen for 30 min and the polymerization was initiated by a VIS-light-lamp. The polymerization was stopped after 24 h. Subsequently, the polymer was dialyzed against deionized water (Spectra/Por 3500 Da). Finally, the sample was freeze-dried and a white solid (357 mg, M_n= 67200 g · mol^{-1}, $Đ$ = 1.3) was obtained.

Synthesis of high molar mass poly(*N,N*-diethylacrylamide)-*b*-poly(4-acryloylmorpholine) ($PDEA_{1850}$-*b*-PAM_{1380})

Destabilized AM (300 mg, 2.1 mmol, 4883 eq.), $PDEA_{1850}$ (100 mg, 0.00043 mmol, 1.0 eq.) and acetate buffer (0.7 mL, 0.2 M, pH=5) were mixed together with a stirring bar in a glass vial (14 mL) and sealed with a septum. The solution was bubbled with nitrogen for 30 min and the polymerization was initiated by a VIS-light-lamp. The polymerization was stopped after 24 h. Subsequently, the polymer was dialysed against deionized water (Spectra/Por 3500 Da). Finally, the sample was freeze-dried and a white solid (320 mg, M_n= 403500 g · mol^{-1}, $Đ$ = 1.5) was obtained.

2. Polymer Analytics

SEC Results

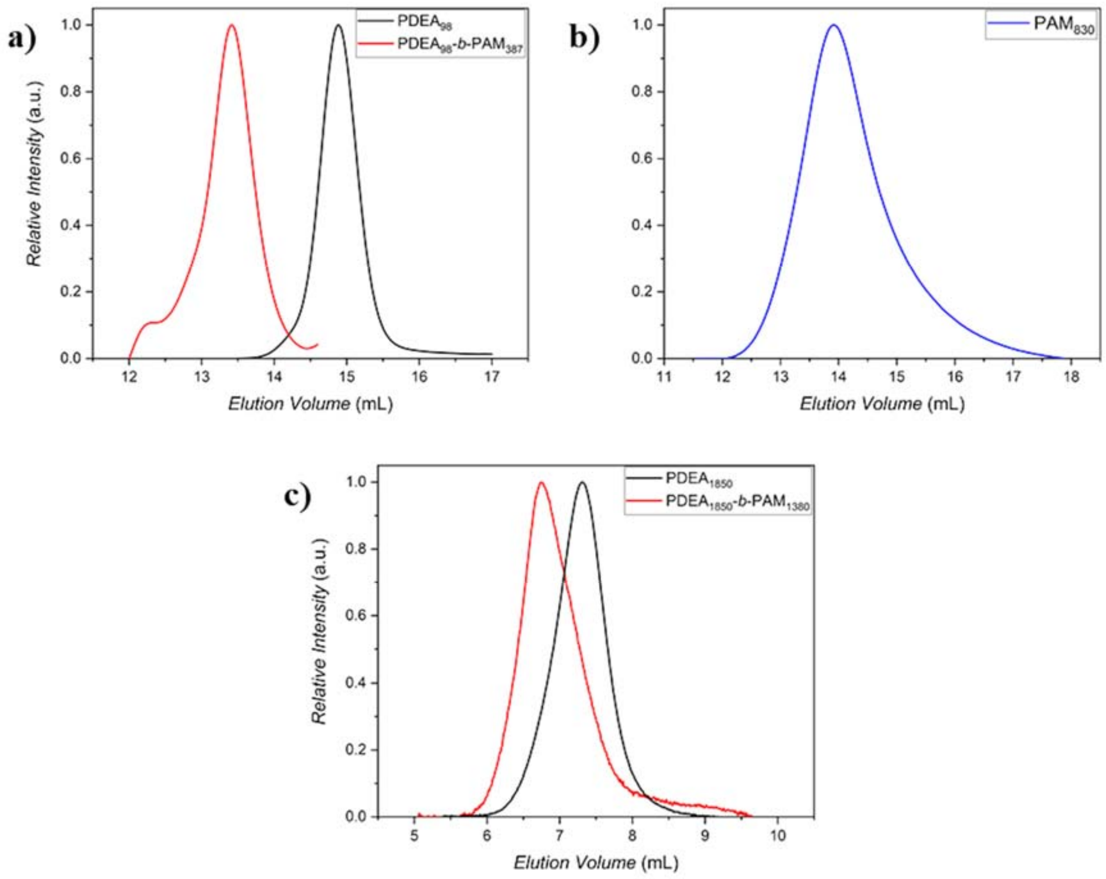

Figure S1. SEC measurement of (a) $PDEA_{98}$ and $PDEA_{98}$-*b*-PAM_{387} in THF, (b) PAM_{830} in NMP against PS calibration and (c) SEC MALS trace of $PDEA_{1850}$ and $PDEA_{1850}$-*b*-PAM_{1380} in THF.

Table S1. Results of SEC measurements of PDEA, PAM and PDEA-*b*-PAM.

Polymer	M_n (kg·mol^{-1})	Đ
**$PDEA_{98}$[a]**	12.4	1.1
**$PDEA_{1850}$[c]**	235.1	1.3
**PAM_{830}[b]**	117.3	1.6
**$PDEA_{98}$-*b*-PAM_{387}[a]**	67.2	1.3
**$PDEA_{1850}$-*b*-PAM_{1380}[c]**	403.5	1.5

[a] in THF against PS standard, [b] in NMP against PS standard, [c] in THF with MALS detection (d*n*/d*c* ($PDEA_{1850}$): 0.090 ± 0.031 mL·g^{-1};
d*n*/d*c*($PDEA_{1850}$-*b*-PAM_{1380}): 0.105 ± 0.038 mL·g^{-1})

NMR Results

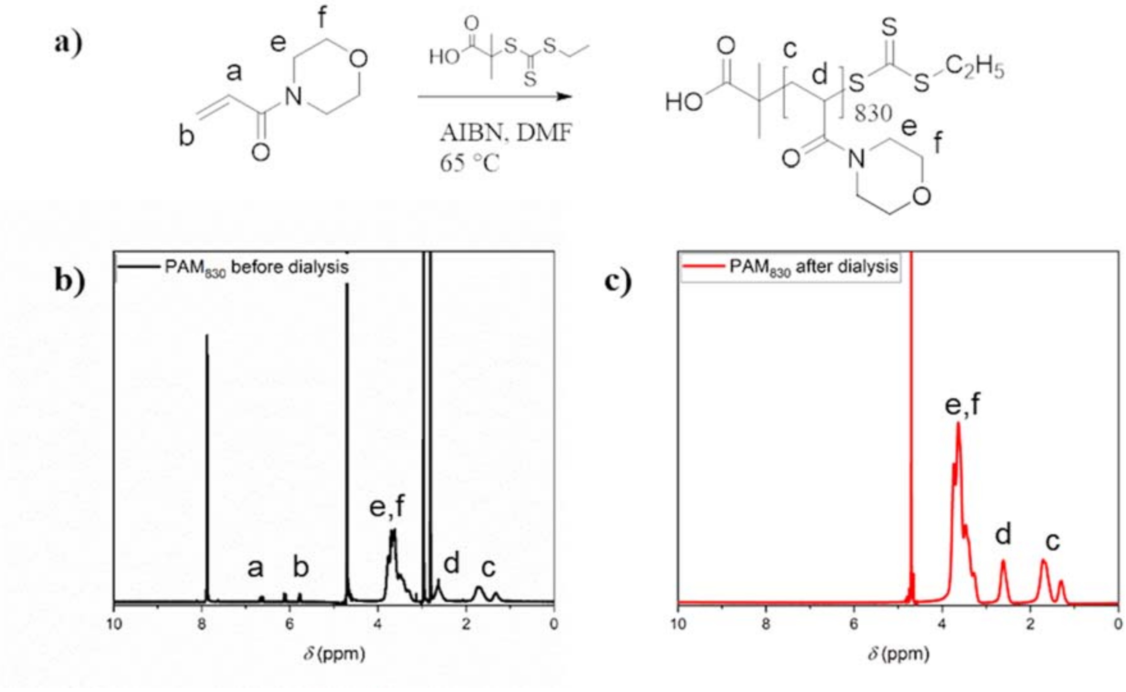

Figure S2. (a) Reaction scheme of the photo induced RAFT-polymerisation of AM, (b and c) ^{1}H-NMR measurement of PAM_{830} in D_2O (b) before dialysis and (c) after dialysis.

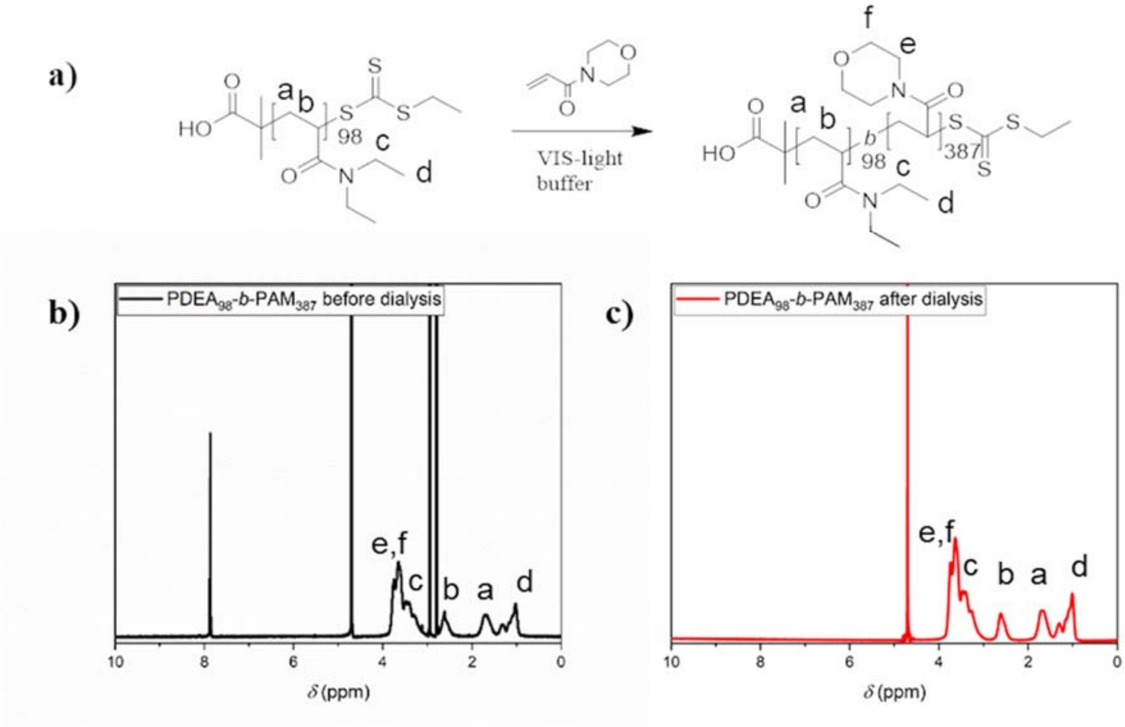

Figure S3. (a) Reaction scheme of the photo induced RAFT-polymerisation for PDEA-*b*-PAM synthesis, (b and c) ^{1}H-NMR measurement of $PDEA_{98}$-*b*-PAM_{387} in D_2O (b) before dialysis and (c) after dialysis.

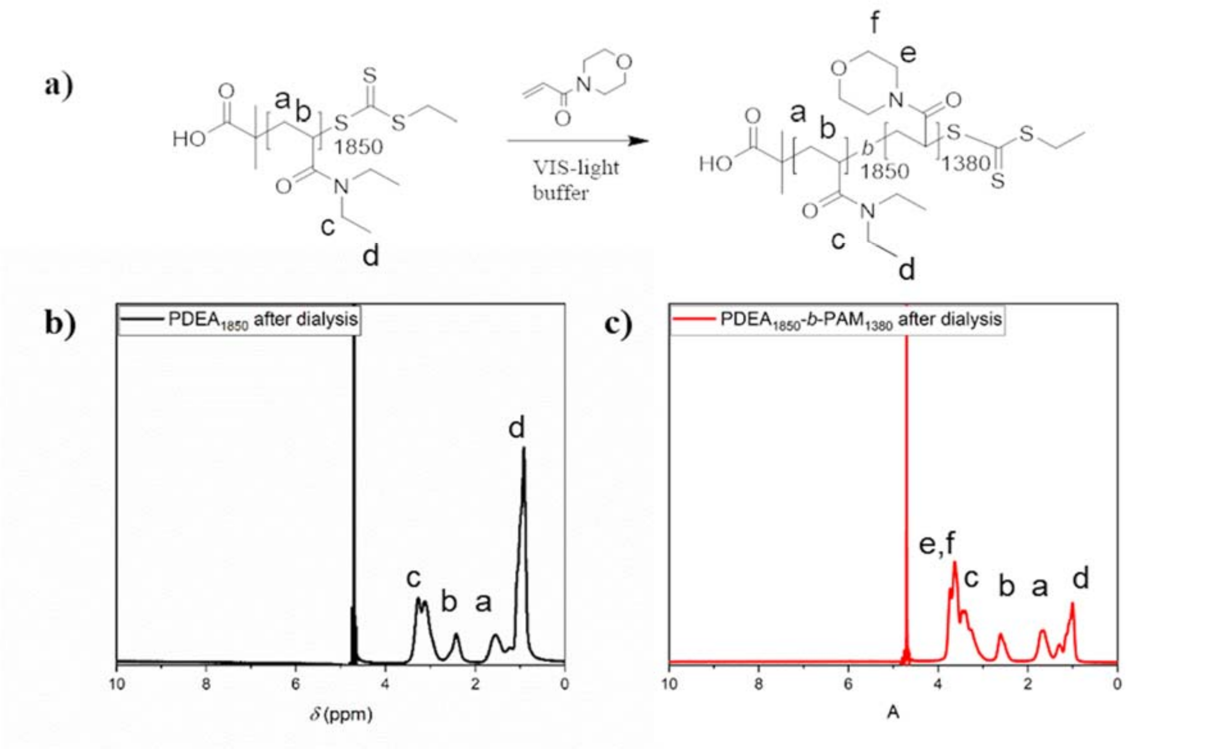

Figure S4. (a) Reaction scheme of the photo induced RAFT-polymerisation of PDEA-*b*-PAM, (b) ^{1}H-NMR measurement of $PDEA_{1850}$ in D_2O and (c) ^{1}H-NMR measurement of $PDEA_{1850}$-*b*-PAM_{1380} after dialysis in D_2O.

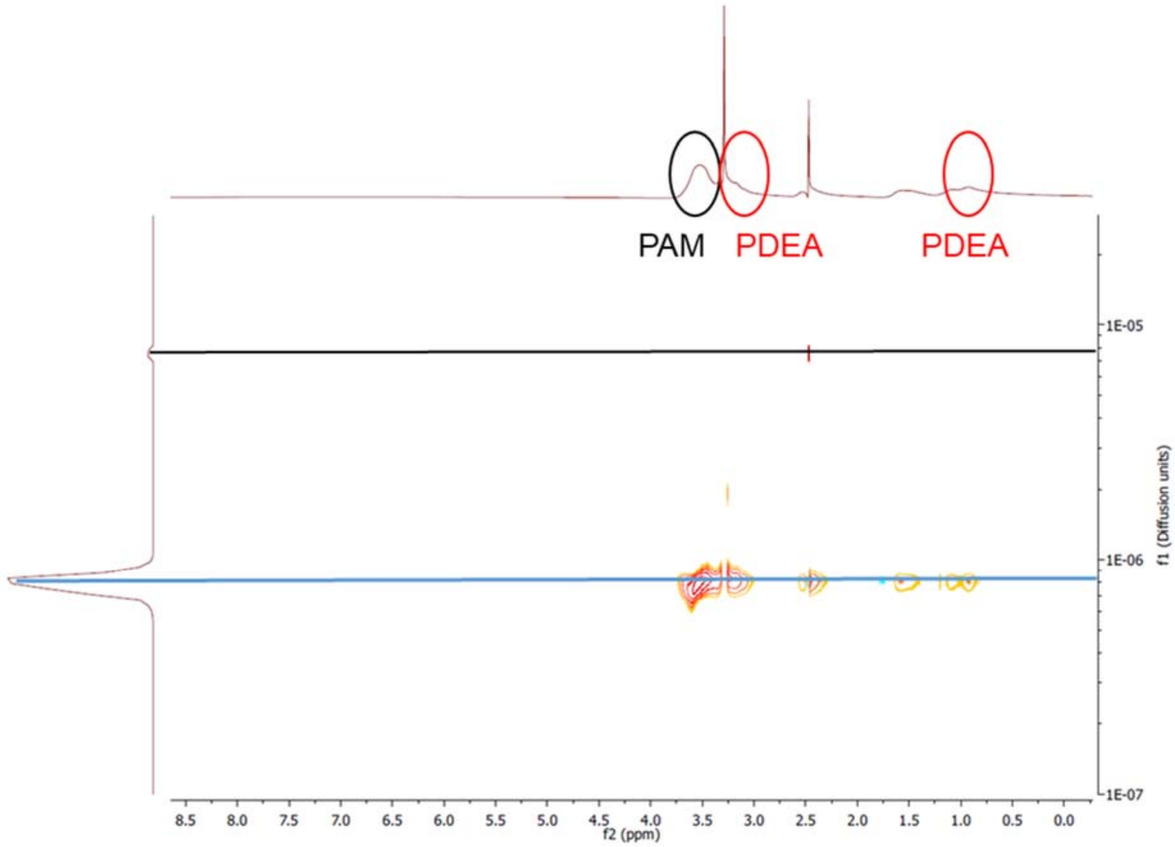

Figure S5. DOSY measurement of $PDEA_{1850}$-*b*-PAM_{1380} (measured in DMSO-d_6) with the diffusion coefficient of DMSO-d_6 (black line) and $PDEA_{1850}$-*b*-PAM_{1380} including all ^{1}H-NMR peaks from all individual blocks (blue line).

DSC Results

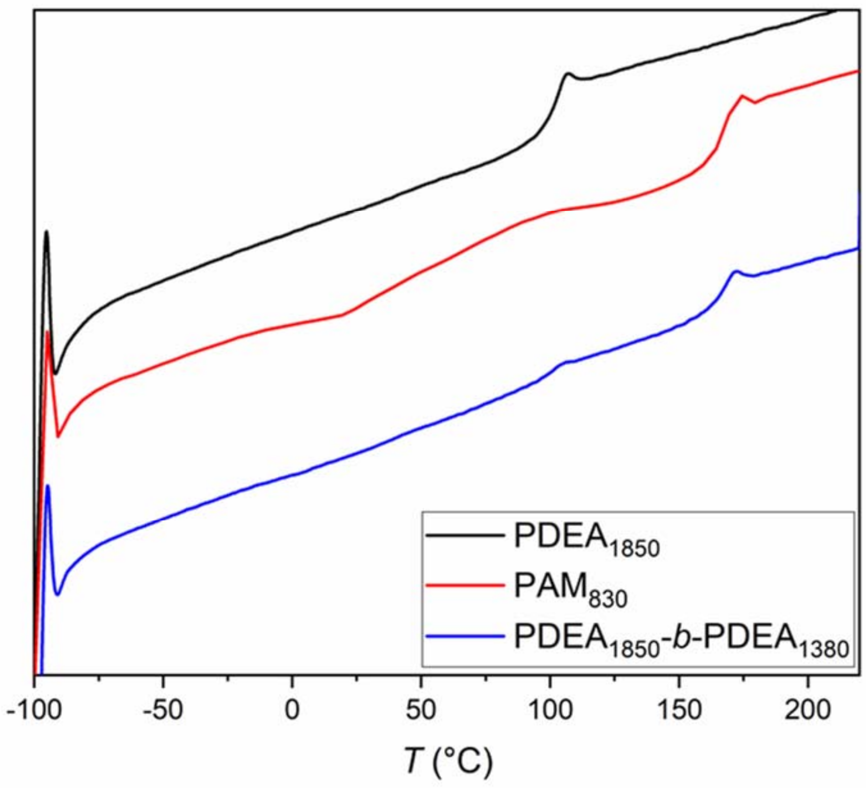

Figure S6. DSC thermograms of $PDEA_{1850}$, PAM_{830} and $PDEA_{1850}$-*b*-PAM_{1380}.

Table S2. Glass transition temperature of $PDEA_{1850}$, PAM_{830} and $PDEA_{1850}$-*b*-PAM_{1380}, according to the inflection point of the main Peaks in the DSC thermogram.

**Polymer**	T_{g1} (°C)	T_{g2} (°C)
**$PDEA_{1850}$**[c]	102.7	-
**PAM_{830}**[b]	169.5	-
**$PDEA_{1850}$-*b*-PAM_{1380}**[c]	103.0	169.6

3. Aggregation Analytics

Cryo-TEM Statistics

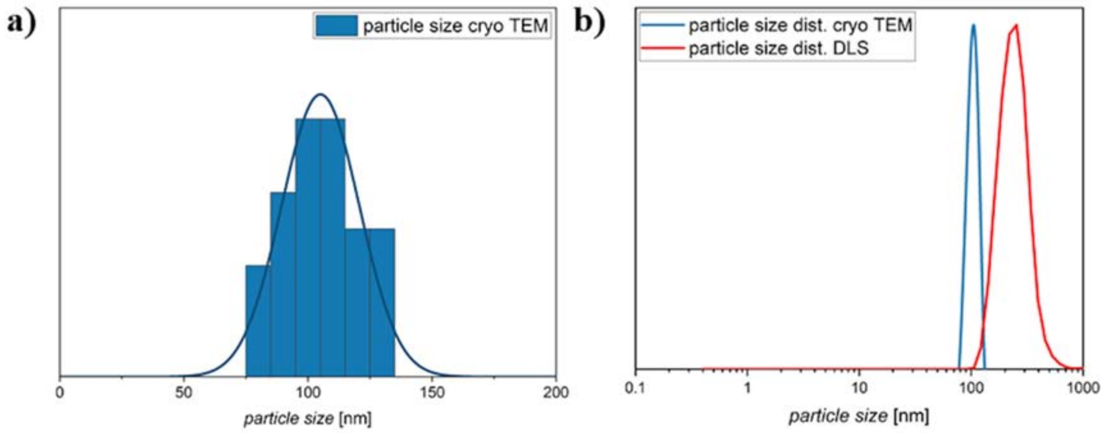

Figure S7. Particle size distribution of PDEA-*b*-PAM at 6 wt% in THF (a) measured over 30 particles in cryo-TEM images including normal distribution and (b) comparison of the particle size distribution measured with cryo-TEM images (blue curve) and DLS (red curve).

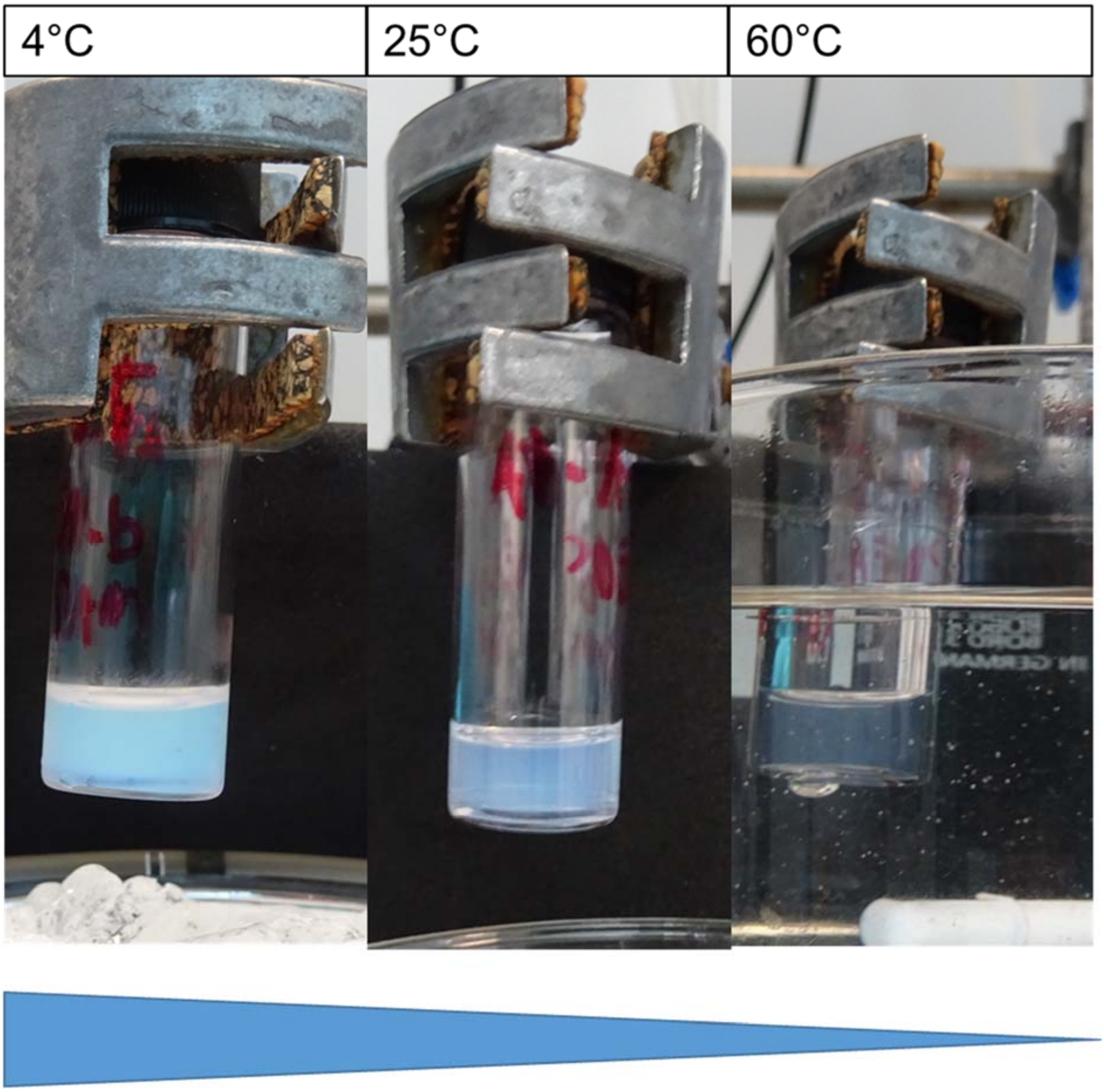

Figure S8. Intensity of the blue coloured dispersion of $PDEA_{1850}$-*b*-PAM_{1380} at 6 wt% in THF depending on the temperature.

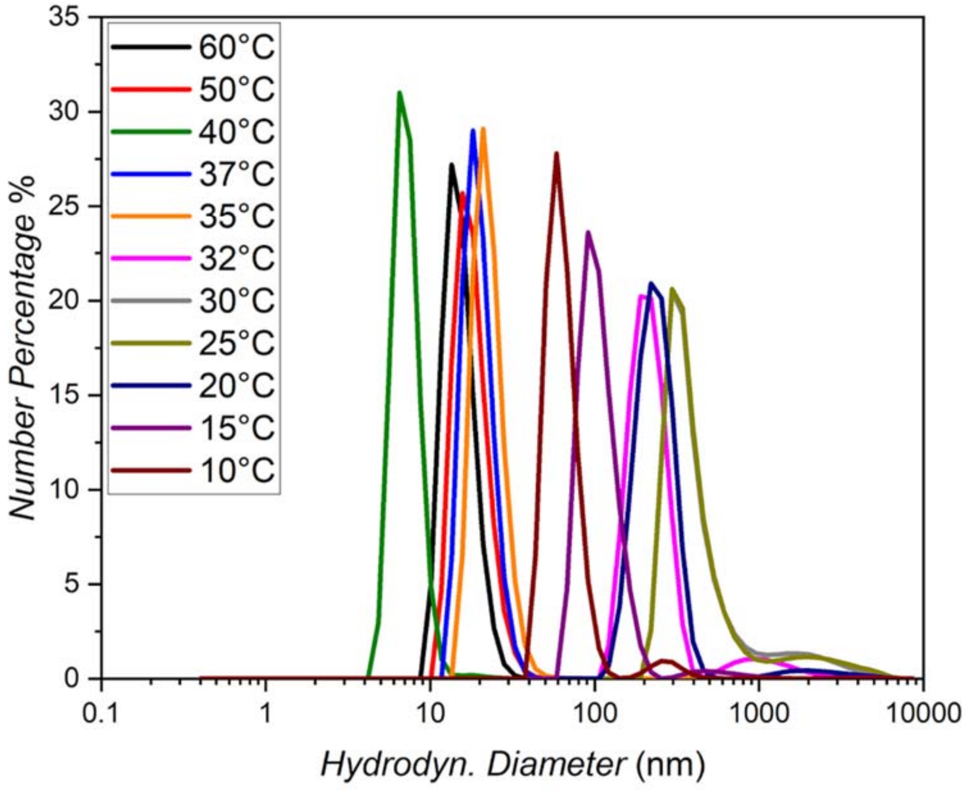

Figure S9. Comparison of number weighted particle size distribution of $PDEA_{1850}$-*b*-PAM_{1380} in THF at 6 wt.% measured via DLS at different temperatures (10-60 °C).

Table S3. Comparison of number weighted particle size distribution of $PDEA_{1850}$-*b*-PAM_{1380} in THF at 6 wt.% measured via DLS at different temperatures (10-60 °C).

***T* (°C)**	**10**	**15**	**20**	**25**	**30**	**32**	**35**	**37**	**40**	**50**	**60**
**Particle size (nm)**	70	91	220	255	255	190	21	18	8	16	14

References

[1] J. Skey, R. K. O’Reilly, Chem. Commun. 2008, 4183.

[2] B. V. K. J. Schmidt, M. Hetzer, H. Ritter, C. Barner-Kowollik, Macromolecules 2011, 44, 7220.
